# CpG oligodeoxynucleotides and pan-serotype inhibitors control neurotropic dengue infection in novel immune competent neonatal mouse model

**DOI:** 10.1080/22221751.2025.2477668

**Published:** 2025-03-10

**Authors:** Mirian Mendoza, Derek D.C. Ireland, Ha-Na Lee, Logan Kelly-Baker, Monica Chowdhury, Daniela Verthelyi, Mohanraj Manangeeswaran

**Affiliations:** Office of Pharmaceutical Quality Research, Division IV, Center for Drug Evaluation and Research, Food and Drug Administration, Silver Spring, MD, USA

**Keywords:** Dengue, mouse model, immunocompetent, immunomodulatory, neurotropic, virus

## Abstract

Dengue virus (DENV) is a growing global public health threat. The lack of symptomatic immune-competent animal models for dengue has hindered the screening and development of effective therapeutics that can be used to control dengue virus replication and thereby control the progression to severe dengue disease. To address this, we established an infection model in neonatal C57BL/6 mice and showed that a systemic Dengue challenge leads to ataxia, seizures, paralysis, and death within 15 days. The virus was found predominantly in the eye and brain where DENV infects neurons but not astrocytes and causes extensive infiltration of macrophages and microglia activation. The response to infection included upregulation of multiple genes linked to interferons (*Ifna, Ifnb, Ifng, Irf7, Irf8, Mx1, Stat1* and *Bst2*), inflammation (*Il6,Tnfa*), complement (*Cfb,C1ra,C2, C3*), cytolysis (*Gzma, Gzmb, Prf1*) consistent with antiviral responses and inflammation together with neuroprotective regulatory signals (*Il27, Il10, and stat2)*. The increased proinflammatory signature was associated with downregulation neurodevelopmental genes *(Calb2, Pvalb, Olig1* and *Olig2)*. We tested the utility of this mouse model by assessing the protection conferred by direct antivirals JNJ-A07 and ST-148 and host-directed antiviral immunomodulatory CpG oligodeoxynucleotide (ODN), alone or in combination against lethal dengue viral infection. The data showed that immunomodulatory CpG ODN and antiviral JNJ-A07 improved the survival of neonatal mice, and protection from lethal neurotropic infection was optimal when treatments were combined. This study suggests that a combination of an effective dengue antiviral along with a host-directed therapeutic may be a useful strategy to protect against dengue virus infections.

## Introduction

Dengue virus (DENV), a member of the flavivirus family, is a mosquito-borne disease that causes dengue fever (DF). DENV is endemic in over 80 countries particularly in tropical and subtropical regions with an estimated annual burden of 390 million infections, of which 96 million can be symptomatic [1,2]. In 2023, there were 6.5 million cases of dengue infection and more than 7300 dengue-related deaths reported [2]. Most symptomatic patients present with flu-like symptoms, including fever, headache, nausea, and joint pain. However, a subgroup of patients can develop dengue haemorrhagic fever (DHF) and/or dengue shock syndrome (DSS) characterized by plasma leakage severe enough to induce shock, respiratory distress, severe bleeding, and/or multi-organ failure, particularly if repeatedly exposed to different serotypes [3,4]. Indeed, approximately 1 in 20 patients develop severe life-threatening Dengue with neurological symptoms [5,6]. Current treatment options are limited and rely on supportive care so there is a critical need for therapeutics and vaccines that can reduce dengue morbidities and lower the infection burden [7].

Upon entry of DENV into the host cell, the positive, single-stranded RNA genome is translated into a single polyprotein that is proteolytically processed to produce three structural proteins, capsid (C), pre-membrane (prM), and envelope (E), and seven nonstructural proteins, NS1, NS2A, NS2B, NS3, NS4A, NS4B, and NS5. There are four genetically related but antigenically distinct DENV serotypes: DENV-1, DENV-2, DENV-3, and DENV-4 of which DENV2 infection is more likely to induce severe disease [8,9]. The innate immune system plays a key role in controlling DENV infections. Nucleic acids in DENV triggers Toll-like receptor (TLR) 3, 7, and 8 in endosomes and retinoic acid-inducible gene I (RIG-I) and melanoma differentiation-associated gene 5 (MDA-5) in the cytoplasm leading to the activation of nuclear factor κ B (NF-κB) and interferon regulatory factors (IRF) 3 or 7 to produce proinflammatory cytokines and type I (α and β) and III (λ) interferons (IFN), respectively [9–12]. The IFN response is thought to be critical, as in the absence of IFN increased viral replication has been observed [13–15], raising the possibility of using prophylactic or therapeutic innate immune modulators that foster interferon responses to treat DENV infection.

Immunomodulatory therapies, including interferons and TLR agonists, are pathogen-agnostic and may be helpful in preventing future emerging and reemerging infectious diseases [16–18]. We previously showed that CpG oligodeoxynucleotides (ODN) can improve the host’s ability to control infections with Tacaribe and Sindbis virus [19–22]. Of note, testing approaches that involve the induction of endogenous production of interferons is not possible in the animal models currently used to study dengue because they rely on the absence of an interferon response to establish productive infections (e.g. AG129 mice) [23]. Current murine models that use immunocompetent C57BL/6 or Balb/c mice have low viremia and are asymptomatic, while those that use IFN-deficient mice result in viremia but no overt clinical manifestation. These mouse models can be used to test antivirals, but their altered immune response limits investigations into host–pathogen interaction and the use of immune modulators as therapeutics. Thus, establishing a symptomatic animal model that is immune competent can improve our understanding of the host response and allow for the testing of immunomodulatory therapeutics or combine immunomodulators with direct-acting antivirals targeting DENV.

It has been previously established that neonatal immunocompetent C57BL/6 mice are susceptible to systemic infection with several viruses, including Zika virus (another member of the flavivirus family), within 1–3 days of birth [24]. These mice develop a symptomatic disease response and can be used to assess immunomodulatory antiviral therapeutics.

Herein, we show that neonatal C57BL/6 mice are susceptible to DENV infection. Using this model of DENV infection, we examined the protection afforded by immunomodulatory therapeutics and DENV antivirals. We selected two antivirals candidates: ST-148 an inhibitor of DENV replication that targets the capsid protein [25,26], and JNJ-A07, a small molecule that targets the DENV nonstructural proteins NS4B and thereby prevents de novo formation of NS3–NS4B complex [27]. CpG ODN 1555, a 12-base phosphorothioate oligonucleotide containing 2 CpG motifs, was previously shown to modulate the cytokine and chemokine response, in the periphery and in the CNS, and protect neonatal mice from lethal neurotropic infection with Sindbis or Tacaribe virus [20,24]. Therefore, we tested the impact of CpG ODN 1555 alone or in combination JNJ-A07 or ST-148 in controlling lethal DENV infection.

## Results

### Susceptibility of neonatal immunocompetent Mice to DENV2

**Figure 1.**
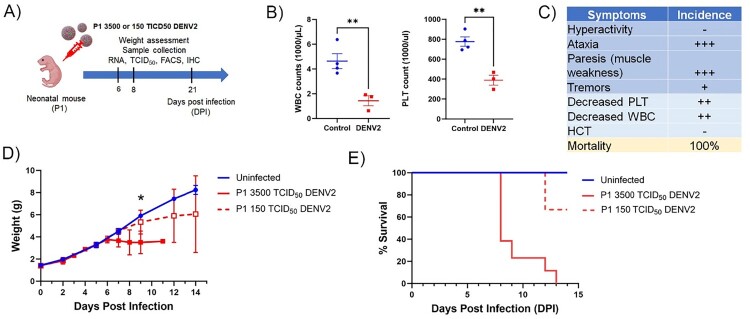
Susceptibility of neonatal immunocompetent mice to DENV2. (A) Experimental scheme created in BioRender.com/o93c834. B6 wildtype mice were subcutaneously infected with DENV2 24 h after birth. (B) White blood cell (WBC) and platelet (PLT) counts were determined in control and DENV2-infected mice at 8 dpi. (C) Mice were monitored for signs of disease, mortality over time and scored from normal (0, −/+) to severe (3, +++). (D) Mice were monitored for weight loss for 3500 TCID_50_ DENV2 infected (*n* = 10), 150 TCID_50_ DENV2 infected (*n* = 3), and uninfected (*n* = 3), and mortality (E) of WT neonatal B6 mice infected with 3500 TCID_50_ DENV2 (*n* = 13), 150 TCID_50_ DENV2 (*n* = 3), and uninfected (*n* = 6). Graphs show mean ± SD; *denotes the weight difference between DENV2 and uninfected mice at 9DPI (*p* < 0.05) as measured by 2-tailed unpaired Student’s *t* test.

**Figure 2.**
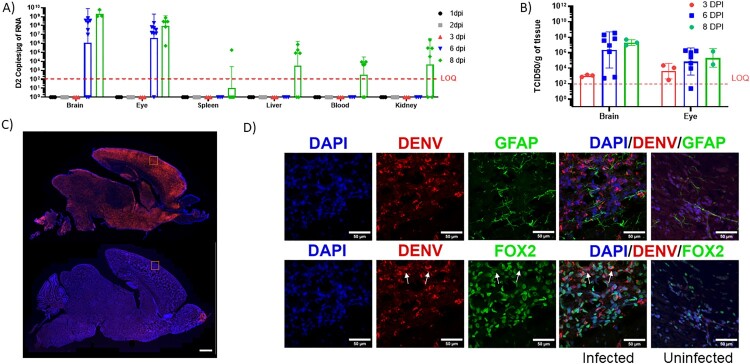
Viral load of DENV2 in B6 WT neonatal mice infected with DENV2. (A) DENV2 RNA was quantified in the CNS and peripheral organs of infected B6 WT mice at 1, 2, 3, 6 and 8 dpi (*N* = 3–6 per group) using quantitative real-time PCR. Values are presented as the number of viral RNA copies/mg of RNA. (B) Viral loads in the brain and eyes of DENV2-infected mice were evaluated by TCID50 at 3, 6 and 8 dpi. Virus-infected cells in the brain of B6 WT mice infected with DENV2 and uninfected control. (C) IF-IHC staining of brain of B6 WT uninfected (bottom) and infected (top) mice with DENV2 (8 dpi). DENV2 virus labelled with 4G2 ab (orange). Scale bar: 1 mm. The scale bar is the same for all figures. (D) Confocal imaging of neurons (Fox2, green), astrocytes (GFAP, green), and DENV2 virus (4G2 ab, red) staining in the cortex of infected or uninfected mice. Arrows indicate infected neurons. Scale bar: 50 μm. The scale bar is the same for all figures.

Previous studies have shown that systemic viral challenges in neonatal C57BL/6 mice before P3 result in lethal neurotropic infections and the clinical outcome and viral load can be modulated using innate immune modulators [19,20,24,28]. To investigate whether neonatal immunocompetent C57BL/6J mice are susceptible to infection with DENV, P5 pups were inoculated subcutaneously with 2860 TCID50 of DENV2 (New Guinea C Strain). Mice challenged at P5 showed resistance to DENV2 infection without any lethality (Supplementary Figure 1). Upon SC challenge of P1 mice with 2860 TCID50of DENV2, fewer than 50% of the mice succumbed to disease (Supplementary Figure 1). On the other hand, newborn pups inoculated subcutaneously with 3500 TCID_50_ of DENV2 at P1, as shown in Figure 1(A), stopped gaining weight (Figure 1(D)) and developed signs of severe neurological disease (Figure 1(C)) that started with tremors, widened stance, and ataxia, progressed to paresis or paralysis by day 8, and succumbed to disease by 8–13 dpi (Figure 1(E)). Moreover, as observed in DENV-infected patients [29], all DENV2-infected mice showed a reduction in white blood cell and platelet counts, indicating leukopenia and thrombocytopenia (Figure 1(B,C)). Although P1 mice SC challenged with a lower dose of DENV2 did not succumb to disease with 100% lethality, the symptoms and disease progression were similar to those of mice receiving the full dose (Figure 1(D,E)) so a challenge of 3500 TCID_50_ and infection at P1 was chosen for subsequent studies. As shown in Figure 2, high titres of DENV2 RNA were evident in the brain and eye of the infected mice by 6 DPI, while the levels in blood, liver, and spleen only reached detectable levels by 8DPI indicating that the virus primarily infects the CNS (Figure 2(A)). TCID_50_ assay confirmed the presence of high titres of replicating infectious virus in the eyes and brains at 3, 6 and 8 DPI (Figure 2(B)). Together, these findings suggest that the neonatal B6-WT immunocompetent mouse model develops a lethal neurotropic infection when challenged with DENV.

**Figure 3.**
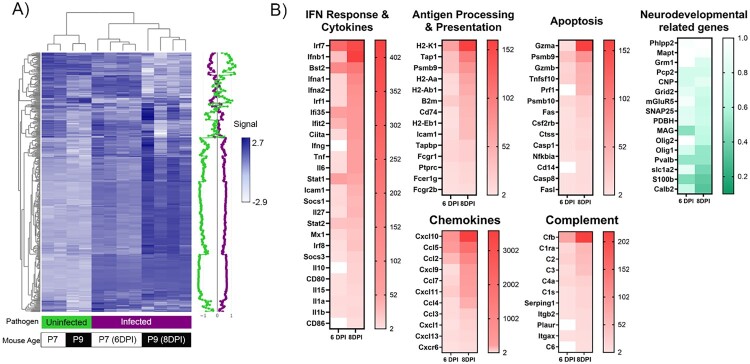
Gene expression in the CNS in response to DENV2 infection. (A) Nanostring mouse immunology panel. Heatmap shows gene expression (normalized log-scaled counts) in the brain DENV2 infected (purple) and uninfected age-matched control (green) at 6 dpi (white) and 8 dpi (black). Each column represents an individual mouse (*n* = 2–4/group) and the green and purple lines on the right depict the average normalized gene expression. Both Genes and Mice are clustered based on distances between the samples and clusters, and the similarity is shown by the respective dendrograms. (B) Heatmaps depict the geometric mean change in gene expression in brain tissue of DENV2-infected mice relative to age-matched uninfected mice.

Having established that DENV2 infects the brain, we next investigated virus distribution within the neonatal brain. As shown in Figure 2(C,D) and Supplemental Figure 2, at 8 dpi the distribution of DENV2 was widespread throughout the cortex, midbrain, and hindbrain (Figure 2(C)). Within the cortex, the virus appears to colocalize with Fox2 but not GFAP suggesting that DENV2-infected neurons but not astrocytes. As such, evidence of astrogliosis consistent in the infected brains was likely linked to inflammation in the CNS (Figure 2(D) and Supplementary Figure 3). Concordant with acute DENV2 infection, the CNS showed a progressive increase in the expression of mRNA for genes linked to type I and II interferons *(Ifnb1, Ifna1, Ifna2, Ifng, Irf7, Bst-2, and Ifi35*), complement (*Cfb, C2 and C1ra)*, cytolytic processes (*Gzma, Gzmb, Prf1, Psmb9&10,* and *Fas)*, antigen processing and presentation (*Tap1*, *Psmb9*, *CD74, H2-K1, H2-Aa, H2-Ab1 and H2-Eb1*), as well as proinflammatory chemokines and cytokines (*Tnf, Il6, Ccl2, Ccl5, Ccl7, Cxcl11* and *Cxcl10*) (Figure 3). Concordant with the increased inflammatory signal, there was a downregulation in mRNA expression of genes linked to neurodevelopment and myelination, neurotransmitter regulation, and psychiatric disorders including calcium binding *Calb2* and *S100b*, as well as *SNAP25*, *Pvalb*, MAG, *Olig1, and Olig2* (Figure 3).

**Figure 4.**
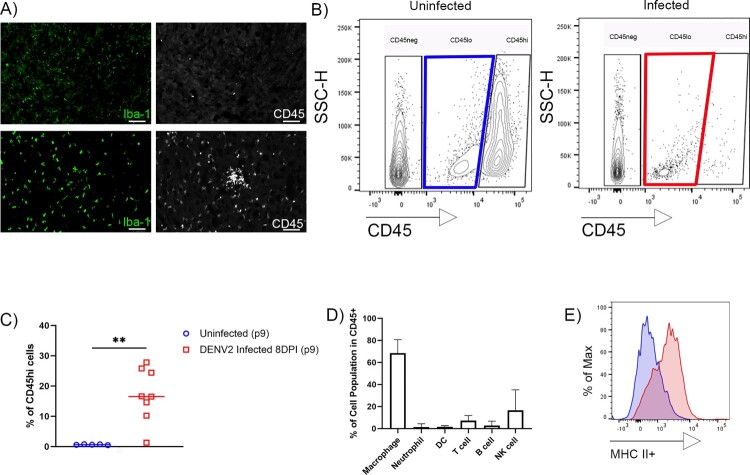
Immune cells infiltrating the CNS in response to DENV2 infection. (A) IF-IHC staining of the brain of B6 WT uninfected (top) and infected (bottom) with DENV2 (8 dpi). Activated microglia (Iba-1, green) and infiltrating immune cells (CD45+, white) are present in the brains of infected mice. Scale bar: 100μm. (B) Flow cytometry was performed on cells isolated from the CNS (*n* = 8) of DENV2-infected B6 WT at 8 dpi. Live cells were gated based on CD45 expression. (C) Percent of CD45^high^ cells in total live cells. (D) Phenotype of CD45^high^ infiltrating cells in CNS. (E) MHCII expression in CD45^low^ microglial cells from the CNS of mice infected with DENV2 (red) compared to uninfected age-matched control (blue).

The increase in mRNA expression for MHC-linked genes (Figure 3(B)) suggested the presence of activated microglia and/or macrophage infiltration in the infected brains. Immunohistochemistry (IHC) analysis showed an increase in Iba-1+ activated microglia and CD45^+^ infiltrating cells distributed throughout the cortex, midbrain, and cerebellum (Figure 4(A) and Supplementary Figure 2). Flow cytometry analysis confirmed that the CNS of DENV2-infected B6 pups have a marked increase in infiltrating CD45^high^ cells (Figure 4(B,C)) particularly macrophages and NK cells (Figure 4(D)), which was consistent with the observed increases in the expression of genes linked to inflammation, cytotoxicity, and innate immune activation (Figure 3(B)). Additionally, as shown in Figure 4(E), CD45^low^ microglia in the brains of B6 infected mice with DENV2 showed increased expression of MHC class II (MHCII), a marker of activation and consistent with the expression of IFNg in the CNS, relative to age-matched uninfected WT mice control. Together these data establish that neonatal B6-WT immunocompetent mice develop a lethal neurotropic infection when challenged with DENV. Although the observed pathology does not recapitulate the haemorrhages or organ failure in peripheral sites (e.g. liver, lung and kidney) observed in patients with severe dengue symptoms [30], the challenged mice develop a symptomatic meningoencephalitis with high viral titres in the brain, as well as thrombocytopenia, suggesting that the mouse model could be useful in the evaluation of potential therapeutics for DENV virus infection.

### Assessment of anti-dengue therapeutics

**Figure 5.**
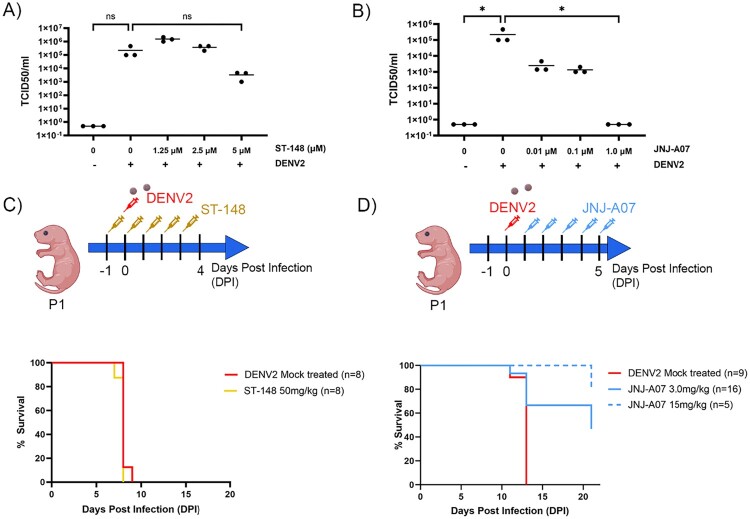
Anti-viral JNJ-A07 improves the survival rate of DENV-infected mice but antiviral ST-148 does not. (A) ST-148 reduces viral load in Vero cells challenged with DENV2. VERO E6 cells were pretreated with ST-148 2 h before challenge with DENV2 (MOI 0.1). (B) VERO E6 cells were treated with JNJ-A07 2 h after challenge with DENV2 (MOI 1). (C) Schematic of experimental design created in BioRender.com/o93c834. P0 WT B6 mice were treated with ST-148 (50 mg/kg) 24 h before challenge with DENV2 and received four additional doses of ST-148 every 24 h thereafter. Survival curve of mice treated with ST-148 and challenged with DENV2. (D) Schematic of experimental design. P0 WT B6 mice were treated with JNJ-A07 i.p. 24 h after infection with a lethal dose of DENV2 and received four additional doses of JNJ-A07 every 24 h thereafter. Survival curve of mice treated with JNJ-A07at 15 mg/kg (dashed blue line) and 3 mg/kg (solid blue line) 24 h after challenge with DENV2.

To date, the selection of antiviral therapeutics has been largely based on their ability to reduce viral replication *in vitro* and reduce viral burden in immunocompromised animal models [31]. Most of the animal models used including non-human primates lack clinical manifestation of dengue virus disease symptoms. Overall, there is a need to develop tools that can show clinical manifestations of dengue disease and can be used to assess the impact of therapeutics, including immune response modulators, on disease progression because (1) *in vitro* neutralization often fails to predict *in vivo* efficacy, (2) immunocompromised models cannot be used to assess immune modulators, and (3) viral loads do not always correlate with disease severity [32]. To test whether or not the neonatal mouse model could be used to assess the effectiveness of anti-dengue therapeutic candidates, we selected ST-148 and JNJ-A07, which were reported to reduce viral replication in vitro [26,27]. As shown in Figure 5(A,B), both antivirals reduced the TCID_50_ titres of DENV2 in VERO E6 cells, albeit at different doses. To determine whether ST-148 and JNJ-A07 would protect the neonatal C57BL/6 mice, we administered a dose that was not toxic for neonates (50 mg/kg) of ST-148 starting at P0, 24 h prior to challenge and continued treatment daily for a total of 5 days (Figure 5(C)). As shown in Figure 5(C), ST-148 administration failed to improve survival in our model. In contrast, mice that received JNJ-A07 (3 or 15 mg/kg IP for 5 days) starting 24 h post-challenge (Figure 5(D)) showed improved survival. Mice that received 15 mg/kg had a survival rate of 80% (4/5) while those that received 3 mg/kg had a survival rate of 44% (7/16; Figure 5(D)). Importantly, among mice that survived DENV infection, there was no detectable viral RNA at the time of weaning (Supplementary Figure 4).

**Figure 6.**
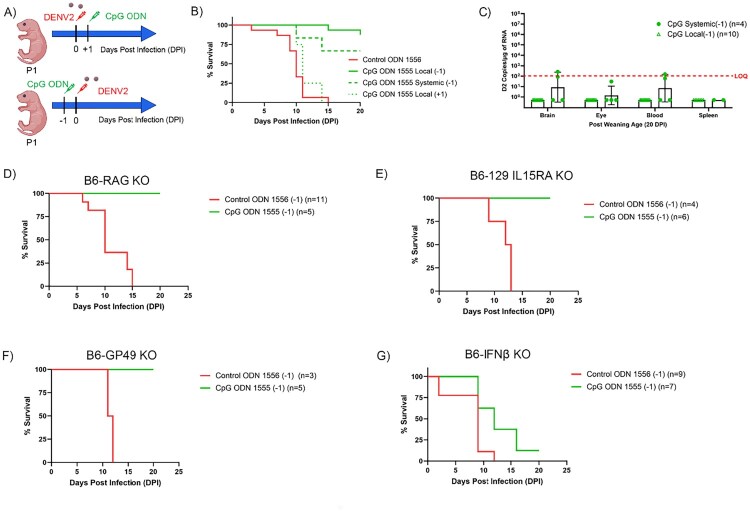
CpG ODN improves the survival rate of DENV- infected mice. (A) Schematic of experimental design created in BioRender.com/o93c834. Top: P0 WT B6 mice were treated with CPG ODN (50ug) subcutaneously and after 24 h infected with a lethal dose of DENV2. Bottom: P1 WT W6 mice were infected with a lethal dose of DENV2 and after 24 h mice were treated with CpG ODN (50ug) subcutaneously. (B) Survival curve of mice treated with control ODN 1556 (50 mg) (*n* = 11), CpG ODN 1555 (50 mg) locally (green solid line; *n* = 16) and systemically 24 h before challenge with DENV2 (green dashed line; *n* = 6). Mice were treated locally with CpG ODN 1555 (50 mg) (green dotted line; *n* = 8) 24 h after challenge with DENV2. (C) DENV2 RNA levels in the CNS and peripheral organs of infected B6 WT mice that received CpG ODN treatment locally (green triangle) and systematically (solid green circle) 24 h before challenge with DENV2 and survived. (D–G) Survival curve of B6-RAG KO (D), B6-129 IL15RA KO (E), B6-GP49KO (F), and B6-IFNbKO (G) mice treated with CpG ODN (50 µg) locally 24 h before challenge with DENV2.

Immunomodulatory drugs such as CpG ODN have been shown to improve outcomes in several infection models including alphavirus and arenavirus [19,20]. Notably, the antiviral activity of immunomodulatory therapeutics like CpG ODN cannot be tested in Vero cells as these do not express TLRs or produce interferons. However, because neonatal C57BL/6 mice have intact immune systems, we could assess whether or not a single dose of CpG ODN administered before or after challenge could improve the outcome in mice challenged with DENV2. As shown in Figure 6(A,B), mice that received 50 μg of CpG ODN 1555 24 h before the challenge survived the infection. Of note, those that were treated 24 h after the challenge did not survive. While survival improved for both routes (*p* < 0.001), the survival rate was higher in mice where the CpG ODN were administered SC at the site of viral challenge (88%) than when the injection was administered SC at a distal site (67%). Notably, a few mice that had received the systemic treatment had trace levels of residual viral RNA at weaning, while those that had been challenged at the site of treatment did not (Figure 6(C)). Together, these studies indicated that the model could be used to compare the impact of antiviral therapeutic regimens on DENV disease progression.

### CpG ODN-mediated protection requires type I IFN response, but not functional NK, T or B cells

CpG ODNs trigger an immunomodulatory cascade resulting in the activation of B and T lymphocytes, natural killer cells, monocytes, macrophages, and dendritic cells [17,33]. The resulting increase in proinflammatory cytokine levels, antigen-specific antibodies and cytotoxic T cells can improve the response of an organism to a virus [17,33]. To better understand the immunoprotective effect of the CpG ODN against lethal DENV2 infection, we first explored whether the protection required functional lymphocytes by challenging B6-RAG KO mice with DENV2 at P1. While untreated B6-RAG KO mice died within 2 weeks of DENV2 challenge, CpG ODN treatment conferred 100% protection against lethal DENV2 suggesting that neither B nor T cells play a critical role in CpG ODN-mediated protection against DENV2 (Figure 6(D)). This was consistent with previous reports of protection in Sindbis infected mice [19]. Next, we explored whether the protection conferred by CpG ODN requires functional NK cells. Inconsistent with the previous finding [34], pretreatment with CpG ODN conferred 100% protection against lethal DENV2 infection in B6-129 IL15RA KO suggesting that NK cells were not critical for the protection mediated by CpG ODN (Figure 6(E)). Moreover, mice lacking glycoprotein 49B (gp49B), which were known to have impairment in the development or activation of NK cells during viral infection [34], were also protected by pretreatment with CpG ODN confirming that NK cells did not play a critical role in CpG ODN-mediated protection (Figure 6(F)). Lastly, it was known that CpG ODN can induce type I IFN responses, so we tested whether IFNβ KO mice challenged with DENV2 could be protected by CpG ODN. As shown in Figure 6(G), IFNβ KO mice pre-treated with CPG ODN had a 12% percent survival suggesting that type I IFN responses were critical in CpG ODN-mediated protection against DENV2 disease.

### Combination of antiviral and immunomodulatory therapeutics

**Figure 7.**
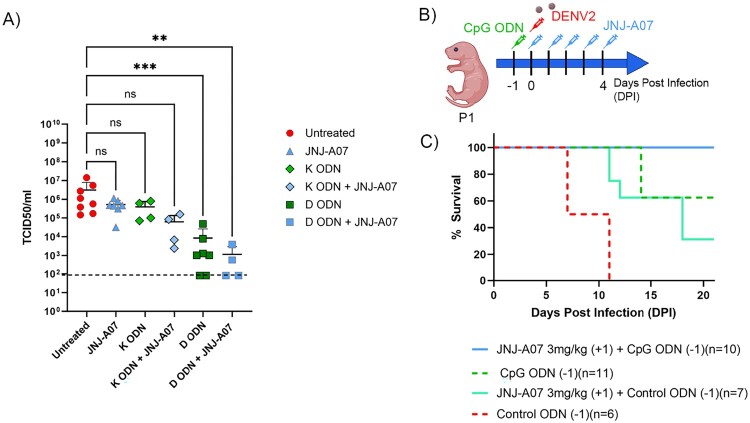
Combination therapy of JNJ-A07 and CpG ODN reduces DENV viral replication on VERO E6 cells and improves the survival rate of DENV-infected mice. (A) Conditioned media from PBMCs stimulated with CpG ODN K3 (diamond) and D35 (square) and JNJ-A07 (blue) at 0.01 μM were added to VERO E6 cells infected with DENV2 at an MOI of 0.1. (B) Schematic of experimental design created in BioRender.com/o93c834. P0 B6 WT mice were treated with CpG ODN (50 µg) systematically 24 h before challenge with DENV2 and received JNJ-A07 at 3 mg/kg on day P1 of life and received four additional doses of JNJ-A07. (C) Survival curve of mice treated with monotherapies of CpG ODN, JNJ-A07 and a combination of both.

Given that the clinical success of antivirals alone for DENV so far has been limited, based on the data observed in this work, a treatment strategy that combines direct-acting antivirals with immune modulators could potentially improve DENV disease management. Because CpG ODN and JNJ-A07 treatment improved survival, we utilized suboptimal regimens for both to determine whether or not they could have additive effects. As shown in Figure 7(B), mice received systemic CpG ODN at P0 (day −1), were infected at P1 (day 0) and then treated with five daily doses of JNJ-A07 at 3 mg/kg starting at the day of challenge. The mice that received both treatments showed 100% survival as compared to 30–40% with the JNJ-A07 alone or 60% with CpG ODN (IP) alone (Figure 7(C)).

It is well established that ODN with CpG motifs that stimulate murine TLR9 are not suited for optimal activation of human TLR9-bearing cells [35]. Indeed, different CpG ODN sequences elicit distinct effects in human cells: CpG ODN type D (or A) are known to selectively stimulate pDCs to induce high levels of type I interferons while CpG ODN type K (or B), which are closer in structure to mouse CpG ODN 1555, trigger increased levels of proinflammatory IL-6 and high titres of antigen-specific antibodies [36]. Directly testing the antiviral effects of CpG ODN on VERO E6 cells is not possible because they do not express TLR9. Moreover, the protective effect of CpG ODN against DENV appears to be mediated by type I IFNs, which are not produced by VERO E6 cells. Therefore, to assess whether the CpG ODN that triggers human TLR9 signalling can accelerate dengue clearance, we collected the supernatant of human PBMC for 3 days with CpG ODN type K or type D, and then overlayed the resulting supernatants on infected VERO E6 cells. As shown in Figure 7A, supernatants from PBMC treated with D-ODN, which induces type I IFNs, reduced the viral titres in VERO E6 cells, while those treated with K-ODN that induces primarily B cell activation and proinflammatory responses did not (Figure 7(A)). We then used the same strategy to determine whether the supernatants of human PBMC treated with D-ODN would improve the viral clearance when combined with a suboptimal concentration of JNJ-A07. As shown in Figure 7(C), combination of JNJ-A07 and supernatants from human PBMC treated with D-ODN- improved viral clearance in DENV2-infected VERO E6 cells, suggesting that the combination of both products may yield better protection than either product individually. Together, these results demonstrate that the neonatal mouse model can have clinical manifestations of dengue virus infection symptoms and be used to assess the ability of direct-acting antivirals or host-directed antivirals or their combination to combat DENV. Results from the current study suggest that combination therapies may be more effective against DENV.

## Discussion

To date, DENV treatment development has been limited by the lack of immunoreplete animal models that recapitulate clinical manifestations of human disease symptoms. For example, in non-human primates, DENV causes viremia (NHPs) but no signs of severe disease [37]. In addition, mice, guinea pigs, hamsters, and rats are resistant to DENV infection and only when challenged with very high doses (e.g. 10^8^ plaque-forming units [PFU]) of an adapted DENV-2 strain 16,681 will adult C57BL/6 mice develop a mild hepatitis or thrombocytopenia [31]. To overcome the inherent resistance of immunocompetent mice to DENV infection, researchers have used animals with deficiencies in innate host defense responses; however, because of these deficiencies, these mice cannot be used to assess the immune response to the virus or test therapeutics that could protect from DENV by modulating the innate immune system. The work presented here provides an alternative model as a physiologic challenge with 3500TCID_50_ of DENV2 SC reproducibly causing thrombocytopenia and leukopenia, which are frequently reported clinical manifestations of DENV in mild and severe cases [38]. Indeed, thrombocytopenia and leukopenia are part of the WHO diagnostic criteria for severe Dengue [39]. The present model’s main symptoms are linked to CNS and ocular infections but show no vascular leakage, or gastrointestinal and respiratory involvement. Therefore, although the mouse model can be used to assess viral clearance and the impact of therapeutics on disease progression, inferences on pathogenesis should be considered cautiously.

The studies above show that systemic challenge with DENV2 reproducibly causes a lethal meningoencephalitis in C57BL/6 neonatal mice despite rapid clearance of the virus from peripheral organs. The virus reaches the brain within 3 days of challenge (SC) infecting neurons in broad areas of the cerebrum, including the cortex, midbrain and hind brain but tends to spare the olfactory bulb. Signs of neuropathology include tremors, ataxia, seizures, paralysis and death in 8–13 days. Immunohistochemistry and flow cytometry show extensive astrogliosis and microgliosis as well as macrophage and NK cell infiltration. The cellular infiltration and inflammation are associated with increased mRNA levels of genes associated with type I interferon and inflammatory responses including *IRF7*, *Bst2*, *CXCL10*, and *Stat1* as well as *IL-6* and *TNFa* but not *IL-1* a/b or *IL-12*. The NK cell infiltration is associated with increased mRNA levels of granzyme and perforin. Testing of antiviral candidates showed that JNJ-A07, a pan-serotype DENV inhibitor targeting the NS3-NS4B, impacted on disease progression in a dose-dependent manner. In contrast, treatment with the capsid inhibitor, ST-148, failed to protect at a dose that was not toxic for the mice. Because the mice are immune replete, it was possible to assess whether treatment with CpG ODN alone or together with an antiviral would have an impact on disease progression; administration of a single dose of CpG ODN improved survival of DENV2-infected mice, and its immunoprotective effects appeared to be additive to those of the antiviral, suggesting that a combined strategy may be more effective.

Severe dengue usually occurs in individuals previously infected with DENV or among infants born to previously infected mothers. CNS involvement is reported in fewer than 20% of DENV cases, but includes neurological complications such as Guillain-Barre syndrome, rhabdomyolysis, confusion, transient muscle dysfunctions, myelitis, and neuro-ophthalmic involvement. Importantly, most severe Dengue cases occur in young children [5]. Perinatal Dengue can associate with intracerebral haemorrhages, neurological malformations, and acute focal/disseminated encephalitis/encephalomyelitis [40]. Direct neuroinvasion has been demonstrated by the detection of DENV antigen in the brain by immune-histochemistry in fatal cases of dengue encephalopathy [41]. A subset of DENV-infected individuals go on to develop severe DENV disease that has significantly higher mortality. Critical determinants that lead to the development of severe DENV disease are not clear. Previous studies suggest that the virus enters the CNS via the haematogeneous route, activates endothelial cells to breach the blood–brain barrier (BBB), infects neurons, and induces cytoarchitectural changes. The neonatal C57BL/6 model reported in this study replicates several features of the CNS involvement, including the influx of NK cells and macrophages. In addition, the increase in proinflammatory cytokines and chemokines in the model including *IL-6*, *TNF*, *CCL2*, *CCL5* and *CXCL1* has also been described in human microvascular endothelial cell (MEC) lines infected with purified dengue virus [42].

The increased susceptibility of neonates to viral infections is well established, and intracranial inoculations of neonatal mice have been used extensively to passage viruses [43,44]. Therefore, until the determinants that lead to the progression of severe DENV disease are clear, the model presented in this study can help understand attributes of DENV-mediated encephalopathy and interventions that can control DENV infection and progression to severe DENV disease. Moreover, systemic infections of neonatal mice prior to one week of life resulting in encephalitis have been reported for other flavivirus including Dengue, and Chikungunya as well as for arenavirus Tacaribe or alphavirus Sindbis [19–21,45,46].

All of these models present *s*igns of neuropathology such as tremors, ataxia, seizures, paralysis and death in 8–15 days. Challenges with Zika, however, resulted in meningoencephalitis but the animals survive. The etiology for the increased susceptibility of neonatal mice to viral encephalitis is not fully elucidated. Some studies using human cord blood pDC have suggested that immune cells from neonates have immature responses via pattern recognition receptors [47,48] and reduced interferon responses, while others find that neonatal immune cells yield similar or even increased cytokine responses to TLR agonists both *in vitro* and *in vivo* [49–51]. In our study, the mRNA expression indicated a strong IFN response and disease progression in untreated mice was similar in neonatal C57BL/6 mice, RAG KO mice or IFNβ KO mice (Figure 6) suggesting that the increased susceptibility of neonatal mice was not linked to defective IFN responses or deficiency in B or T cell responses. Alternatively, the increased susceptibility could correspond to an immature BBB that allows the virus to infect the brain. Once in the parenchyma, the abundance of neuron precursors and high frequency of mitotically active immature neurons, active pruning, and differential expression pattern of cellular receptors, may facilitate seeding and expansion within the CNS [52,53]. Interestingly, while the increased susceptibility of neonatal mice to infections of the CNS may be developmental, the tropism of individual viruses within the brain can differ significantly. DENV2, like Sindbis virus and Zika, infects neurons in broad areas of the cerebrum, including the cortex, mid- and hind- brain, but Sindbis infection tends to spare the cerebellum. In contrast, Tacaribe and VSV-EBOV virus infect primarily the cerebellum, sparing the frontal lobes. Moreover, DENV2, like Sindbis virus and VSV-EBOV virus, appears to primarily infect neurons, while Tacaribe tends to infect astrocytes, especially protoplasmic astrocytes in the granular layers [21]. Additionally, DENV2, like Zika virus, infects the eyes of C57BL/6 neonate mice [54]. Viral RNA and infectious viral titres are detectable in the eyes of challenged DENV-infected mice at both 6 and 8 DPI. However, at 3 DPI low viral titres are detectable by TCID_50_ while viral RNA is undetectable by RT–PCR. This observation suggested that viral distribution may not be homogenous in both eyes early in the course of infection, but as the infection progresses, from 3 DPI to 6 DPI, the virus replicates robustly allowing detection of DENV in both eyes of the mouse model. Together, this suggests that the neonatal brain and eyes provide a permissive milieu for viruses to expand, but their tropism is virus-specific. Therefore, neuroinvasion in the case of DENV infection may be due to a unique susceptibility window in neonates and does not represent attributes contributing to the development of severe DENV disease in humans. However, the model represented in this study can help understand attributes of DENV-mediated neurological diseases and can be used to test dengue-acting antivirals (DAA) and host-directed antivirals targeting DENV disease in the periphery and CNS.

Characterization of the encephalitis caused by DENV2 shows that although DENV2 infects neurons, the virus induces extensive astrogliosis and microgliosis as well as macrophage and NK cell infiltration. The cellular infiltration and inflammation are associated with increased mRNA levels of genes associated with type I interferon and inflammatory responses including *IRF7*, *BSt2*, *CXCL10*, and *Stat1* as well as *IL-6*, *TNFa*, and *IFNg* but, interestingly, not IL-1 a/b or IL-12. The increase in mRNA for CCL5, CCL2, CCL7, granzymes and perforin is consistent with the macrophage and NK cell infiltration. Indeed, NK cells may play a key role by facilitating the clearance of DENV-infected cells through apoptosis or antibody-dependent cellular cytotoxicity (ADCC), which could enhance damage to the CNS architecture. Notably, recent studies assessing NK cells in the blood of DENV-infected patients showed an increased frequency of NK cells in patients with mild Dengue (without warning signs), but reduced number in those with warning signs or severe dengue; moreover, those with severe hemorrhagic Dengue appeared to have a skewed repertoire of NK cells [55,56]. Additional studies on characterization of NK cells that infiltrate the CNS of the DENV-infected mice may shed some light on the role of NK cells in DENV2-encephalitis. Lastly, the data show that the infection and consequent inflammation were associated with reduced expression of genes linked to the development and maturation of the central nervous system including *Pvalb*, *calb* and *Snap25*. These findings are in line with previous findings in mice infected with Zika or SARS-CoV-2 [57,58].

Dengue poses a major challenge to global public health; however, currently, there are limited vaccines available and no licensed specific antiviral treatments. While a high viral load does not always correlate with the severity of disease in other models, using this innate immune-competent mouse model, we observe a strong correlation between viral load and disease severity thereby suggesting that an antiviral that reduces viral load will reduce disease severity. Given the existence of four distinct serotypes and the inherent risk of mAbs and antibody-dependent enhancement, small molecule antivirals may be a preferable strategy for the treatment of DENV infection. To test whether our model could be used to assess the protective effect of therapeutics, we tested 2 antivirals that target different stages in the virus life cycle: ST-148, which binds the capsid protein, and JNJ-A07, which blocks the interaction between NS3 and NS4B within the viral replication complex. Both had demonstrated antiviral activity in *in vitro* studies and *in vivo*, in the AG129 model [26,27]. While both reduced the virus load in Vero cells, only JNJ-A07 significantly improved the survival of neonatal mice challenged with DENV2. Different from previously published data, protection was evident despite the mice being treated only once per day to reduce mouse handling, and dosage was limited to 15 mg/kg to maintain an inoculation volume <20 µL (reference for previous data). Indeed, even when the mice received doses as low as 3 mg/kg, JNJ-A07 showed improved survival, particularly when combined with CpG ODN. Differences in the administration of JNJ-A070 vs ST-148 may have contributed to the outcomes reported but these differences were allowed to provide best-case scenario for the therapeutics tested.

Dengue virus was originally confined to tropical and subtropical areas; however, recently outbreaks in Asia and South America show that the virus has spread outside the former geographic area endangering a larger number of people. Moreover, the expanded range of each of the four dengue virus serotypes and the increase in international travel increases the risk for individuals who have been infected previously with one serotype to become infected with another. Therefore, there is a need for inexpensive effective therapeutics that can be easily deployed to at-risk subjects to reduce the incidence of severe Dengue. Development and selection of effective therapies require in vivo systems with an intact immune system that better model host–pathogen interactions. The neonatal C57BL/6 model used in this work replicated some clinical features of DENV2 infection, including lymphopenia and thrombocytopenia as well as the presence of the virus in the CNS. The C57BL/6 DENV model also enabled the testing of different types of therapeutics and innate immune modulators to treat the disease in an animal model with a functional immune system. Overall, the DENV mouse model provides a new tool for identifying drugs and therapeutic combinations of drugs that can treat severe Dengue infections.

When Dengue virus is injected subcutaneously by the Aedes aegypti mosquitos, local dendritic cells, macrophages and monocytes are activated via pathogen-recognizing receptors RIG-I, MDA5 and TLR3 to produce type I and II IFNs. The virus has been shown to interfere with innate immune signalling via different mechanisms, including proteins that bind and degrade STAT2 and STING [59]. Thus, the use of innate immune modulators, particularly those that elicit and/or maintain an interferon response, has been proposed for the treatment of viral infections. Importantly, while activation of the innate immune system can improve viral clearance, excessive or sustained activation can potentially enhance local inflammation, neurotoxicity, and neurodegeneration. Therefore, the availability of animal models where the response of the infected tissue can be assessed is important to understand which immune modulators could potentially improve the disease outcome by therapeutic intervention. CpG ODNs have been proposed as broad-spectrum immune modulators [33,60]. Importantly, despite their immunomodulatory activity, multiple clinical studies indicate that these ODNs have not been linked to autoimmune or autoinflammatory processes and CpG ODNs have been recently licensed as adjuvants for vaccines for hepatitis B and anthrax. In previous studies, we had shown that the administration of CpG ODN modulated the expression of immune markers in the brain of uninfected mice and that treatment with CpG ODN could improve the outcome of infection with Sindbis virus [19]. These studies showed that CpG ODN had the ability to create an antiviral state in the CNS and could control virus infection. Here, our studies confirmed that treatment with CpG ODN could improve the outcome in infected animals. Of note, previous studies had shown that the immunomodulatory effects of CpG ODN extended to the CNS as cells in the CNS, (some of which expressed TLR9) responded by producing chemokines and cytokines *in vitro* and *in vivo* even in the absence of infection. Any intervention that can create an antiviral state in the CNS may help control the progression of DENV infection to severe DENV disease. As previously shown, the immunoprotective effect of CpG ODN did not require B cells or T cells as protection and was complete in RAG KO mice that did not have mature B cells or T cells present (Figure 5). Interestingly, the protection did not involve NK cells, as IL15RA KO and LILRB KO mice, which have a defective NK cell development, were protected by the treatment. However, mice that lacked IFNβ were not protected by CpG ODN suggesting that the observed mechanism of protection was by enhancing the IFN response [19,59,61,62].

Drug resistance is a major hurdle in the development of effective antivirals, especially those directed at RNA viruses. All RNA viruses display error-prone replication strategies resulting in the accumulation of mutations, the emergence of quasi-species and the appearance of resistant strains. One possible strategy to reduce resistance is the deployment of combination therapies that use orthogonal strategies to control the infection. Activation of the host’s innate immune cells along with a DAA may accelerate viral clearance and reduce the risk of developing escape mutations. In Figure 6, we show that treatment with both CpG ODN and JNJ-A07 improved survival, even when each of the treatments was used at subtherapeutic doses or suboptimal routes. The use of suboptimal conditions was needed to show the additive effects of the 2 therapeutics. As expected, the improved protection afforded by CpG ODN appeared to be linked to the capability to induce IFN responses, as CpG ODN type D, but not Type K, improved viral clearance. Together these data suggest that therapeutic strategies that combine antivirals and immune modulators, may provide a more effective therapeutic approach for symptomatic DENV-infected patients.

## Material and methods

### Reagents

Phosphorothioate CpG ODN 1555 (GCTAGACGTTAGCGT) and ODN lacking a CpG motif ODN 1556 were synthesized at the CBER core facility. DMEM, phenol red-free EMEM, 2X EMEM, penicillin streptomycin solution (100×), L-glutamine solution (100×), trypsin (0.25%)-EDTA (1 mM) and HEPES were purchased from Invitrogen. FBS was purchased from (R&D Systems). JNJ-A07 was purchased from MedChem Express and reconstituted in 10%DMSO, 40% PEG300, 5%Tween80 and 45% saline.

### Cell lines and virus

VERO E6 cells were purchased from American Type Culture Collection (ATCC). Dengue virus type 2-New Guinea C strain was obtained from Barry Falgout, OVRR, CBER, FDA. The virus was originally isolated in 1952 from Culex univittatus and had a limited number of passages in suckling mouse brains and tittered using plaque assays. Further passage in VERO E6 cells was carried out to produce stocks used for the experiments described here. Aliquots (10 μL and 1 mL) of this stock were frozen at −80°C and were used for mice infection experiments. Viral titres were determined using the TICD_50_ Assay.

### TCID_50_ assay

DENGUE2 TCID_50_ assay was performed on VERO E6 cells. VERO E6 cells were seeded on 96 well plates to reach 80–90% confluency the next day. Samples were serially diluted and inoculated onto 96 well plates. Current media was removed, and 100 μL was inoculated into each well and 4-8 replicates were performed for each dilution. Five days post-infection, cells were observed under the microscope and wells were marked infected or not based on cytopathic effect (CPE). Tissue Culture Infectious Dose (TCID_50_) values were calculated as previously described [24]. Organs were homogenized in non-supplemented DMEM media and centrifuged at 300 G for 5 min. The supernatant was then serially diluted in non-supplemented DMEM media and 100 µL/well was plated in a 96-well plate seeded with VERO E6 cells. Cytopathic effect was read 5 days after inoculation and TCID_50_ was calculated as previously mentioned.

### Conditioned media

Leukopaks can be processed into human peripheral blood mononuclear cells (PBMCs). PBMCs were seeded in RPMI medium (10% FBS, Na pyruvate and Pen Strep) in 12 well plates at a density of 4 × 106 cells/mL. Cells were stimulated with no CpG, CpG D35 and K3 at a final concentration of 3 µM for 72 h at 37°C. Following stimulation, supernatants were collected and stored at −80°C.

For the combination studies *in vitro*, VERO E6 cells were seeded at 20,000 cells/well and DENV2 was added at 2000 TCID_50_/well for a final MOI of 0.1. Conditioned media was added at 50%, 20%, 10%, 1%, 0.1% and 0.01%. JNJ-A07 was diluted in media and added to the wells for a final concentration of 0.1 µM, 0.01 µM and 0.001 µM. Cells were incubated with treatments for 5 days and supernatants were collected and stored at −80°C. To determine the TCID50 value of each supernatant collected, VERO E6 cells were seeded in 96 well plates. The cleared supernatant was then serially diluted in non-supplemented DMEM media and added at 100 µl/well. CPE was read at 5 days after inoculation and TCID50 was calculated as previously described.

### Mice

C57BL/6 (B6) WT mice were purchased from the Jackson laboratory. *GP49b–/–* (LILRB4-KO) mice on the C57BL/6 background were provided by Eric Long (National Institute of Allergy and Infectious Diseases/NIH, Rockville, Maryland, USA) [63]. All experiments performed with genetically modified mice were from animals purchased from Jackson Laboratory. All mice experiments were performed at the accredited pathogen-free animal facility of the U.S. Food and Drug Administration and all experiments were approved by the Food and Drug Administration's Animal Care and Use Committee.

### Mice infection

Dengue virus type 2 NGC (New Guinea C strain) was used in our study, and was obtained from Barry Falgout, OVRR, CBER, FDA. Neonatal mice were born to native parents and inoculated on day 1 or day 5 of life with 2000PFU or 3500TCID_50_, (20–40 μL volume) by subcutaneous (S.C.) injection. For survival studies, mice were monitored daily, and weighed every other day. Platelet and white blood cell counts were evaluated using the Heska Element HT5 blood analyzer. For assessment of therapeutics, experiments were carried out with neonatal mice and S.C. treatment with 50 μg CpG ODN (10 μL volume) on days 0, or 2 of life and infected subcutaneously (SC, inter-scapular area) with DENV2 (3500TCID_50_, 20 μL–40 μL volume) on day 1 of life. Mice received ST-148 S.C. treatment at 50 mg/kg on days 0, 1, 2, 3, and 4 of live and infected S.C. with DENV2 (3500TCID_50_, 20–40 μL volume) on day 1 of life. Mice received JNJ-A07 I.P. treatment with 15 mg/kg and 3 mg/kg on days 1, 2, 3, 4, 5 of life and infected S.C. with DENV2 (3500TCID_50_, 20–40 μL volume) on day 1 of life. Mock-infected and/or mock-treated mice were used as controls. Mice were monitored daily, and weight was measured every 2–3 days for survival experiments. Animals with hind limb paralysis and not capable of reaching food or the dam were considered moribund and euthanized. On the day of euthanasia, mice were exposed to CO, and perfused with PBS before any tissues were collected. Samples were frozen at −80°C until processed further. Brain sample was sectioned sagittally, and one half was stored in Trizol for RNA analysis and the other half was frozen in media for viral titre analysis or stored in 4% paraformaldehyde (PFA) for immunohistochemistry analysis.

### RT–PCR

Dengue virus RNA levels were measured using quantitative one-step reverse transcriptase PCR to amplify Dengue virus 2 envelope position 1873–2071 (GenBank accession # NC_001474) (ref). Dengue virus RNA transcript levels in the samples were quantified by comparing to a standard curve generated using dilutions of an RNA transcript copy of dengue virus envelope sequence. Data analysis was carried out using ViiA7 RUO software or Expression suite V1.03.

### Flow cytometry

After perfusion with PBS, brain tissues were collected and digested in RPMI 1640 containing 0.1% trypsin plus 0.015% DNase I for 30 min at 37°C with pipetting every 10 min. Cells from brains were resuspended in 10 mL of 30% Percoll (GE Healthcare, now Cytiva) and then underlaid with 1 mL of 70% Percoll. Samples were centrifuged at 800 g at 4°C for 30 min, and cells were collected from the 30%–70% interface, washed in RPMI 1640, and isolated by centrifugation at 400 g at 4°C for 5 min. Isolated cells were counted and then stained with a cocktail of antibodies against various lineage markers, including CD45 (BioLegend, 103149), F4/80 (BioLegend, 123120), CD11b (BioLegend, 101242), CD11c (BioLegend, 117318), Ly6G (BioLegend, 127633), CD3 (BioLegend, 100351), NK1.1 (Invitrogen, 12-5941-82), CD4 (BD Biosciences, 552775), and CD8a (BioLegend, 100747). Flow cytometry data were collected with the LSRFortessa X-20 flow cytometer (BD Biosciences), and the results were analysed using FlowJo software.

### Immunohistochemistry

Brains were harvested after perfusion with PBS, fixed in 4% PFA, mounted on OCT and flash frozen in liquid nitrogen. After more than 24 h in fixative, the half-brains were rinsed with PBS and submerged in 30% sucrose for cryo-protection. After brains sank in 30% sucrose solution, they were embedded in TissueTek O.C.T (Sakura-Finetek). Brain tissues were sectioned sagittally at 400 μm intervals spanning an entire hemisphere using a cryomicrotome, mounted on superfrost+ slides, and frozen at −80°C. To detect DENV2, sections were stained with mouse anti-Flavivirus polyclonal antibody (4G2) and goat anti-rabbit secondary antibody. Fox-2 (Biolegend, San Diego, CA) was used to stain neurons. DAPI was used to stain nuclei and anti-GFAP antibody (Dako, Carpinteria, CA) was used to stain astrocytes. Tissue sections were incubated overnight in a humidified chamber at 4°C with primary antibodies diluted with 1% BSA in PBS with 0.05% Tween-20. The slides were then rinsed in PBS and incubated with the corresponding secondary antibodies, diluted in 1% BSA in PBS with 0.05% Tween-20 (ThermoFisher, Carlsbad, CA) for 120 min at RT. To reduce autofluorescence, slides were treated with the autofluorescence eliminator reagent. All IF-IHC sections were mounted with ProLong Diamond anti-fade mounting media containing DAPI (ThermoFisher, Carlsbad, CA). Fluorescently labelled antibodies were detected at emission wavelengths: 405 (DAPI), 535 (Alexa-fluor 488), 605 (Alexa-fluor 568). Sections were imaged using a Panoramic Digital Slide Scanner. Images were captured using Panoramic Viewer software (3DHistech, Budapest, HUN).

For confocal imaging, sections were imaged using a Zeiss LSM 880 confocal microscope, using 405, 488, and 561 nm excitation lasers. Consistent laser settings were used to acquire images of both uninfected and infected sections. Maximum intensity projections of these Z-stacks were generated using the Zen software. All images were prepared for publication using image J.

### Nanostring

mRNA expression levels of immune mouse genes were determined using a mouse immunology panel (NanoString Technologies) consisting of 561 genes. Isolated RNA (100 ng) from the CNS was mixed with the reporter and capture probes from nCounter Mouse Inflammation v2 Panel at 65°C for 18 h. Afterward, hybridized products were processed, and data was acquired using the Nanostring MAX acquisition system. Data was normalized to internal controls and housekeeping genes as per manufacturer’s instructions. Data were analysed using the nSolver4.0 software (NanoString Technologies) and heatmaps were plotted using GraphPad Prism. Dendograms were generated using in R using the iheatmapr software. Dendrograms were generated in R 4.0 using the iheatmapr package to plot gene values [64,65]. The signal was defined as the log-scaled Counts normalized by the Gene.

### Statistical analysis

Survival experiments were analysed using the low-rank test. Two-tailed unpaired Student *t*-test or 1-way ANOVA with Dunnett’s multiple comparisons was used as appropriate. Statistical analyses were conducted with GraphPad Software. *P* values lower than 0.05 were considered statistically significant.

## Supplementary Material

Supplementary Figures.docx
