# Supplementary figures and images for: CpG oligodeoxynucleotides and pan-serotype inhibitors control neurotropic dengue infection in novel immune competent neonatal mouse model
Source: Emerg Microbes Infect. 2025 Mar 10;14(1):2477668. doi: 10.1080/22221751.2025.2477668 (PMC12269057; doi:10.1080/22221751.2025.2477668)

**
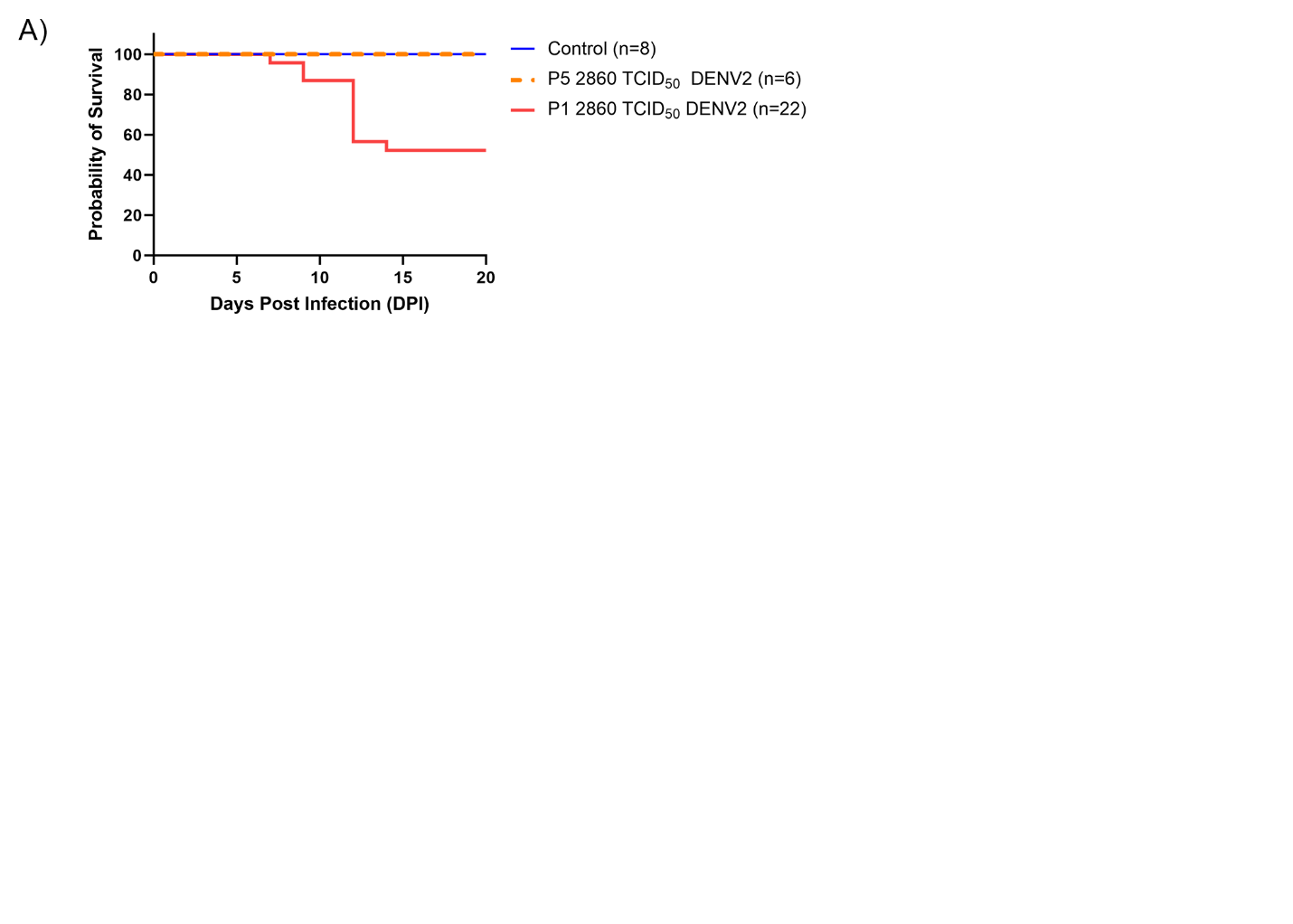
**

**Supplementary Figure 1**

**
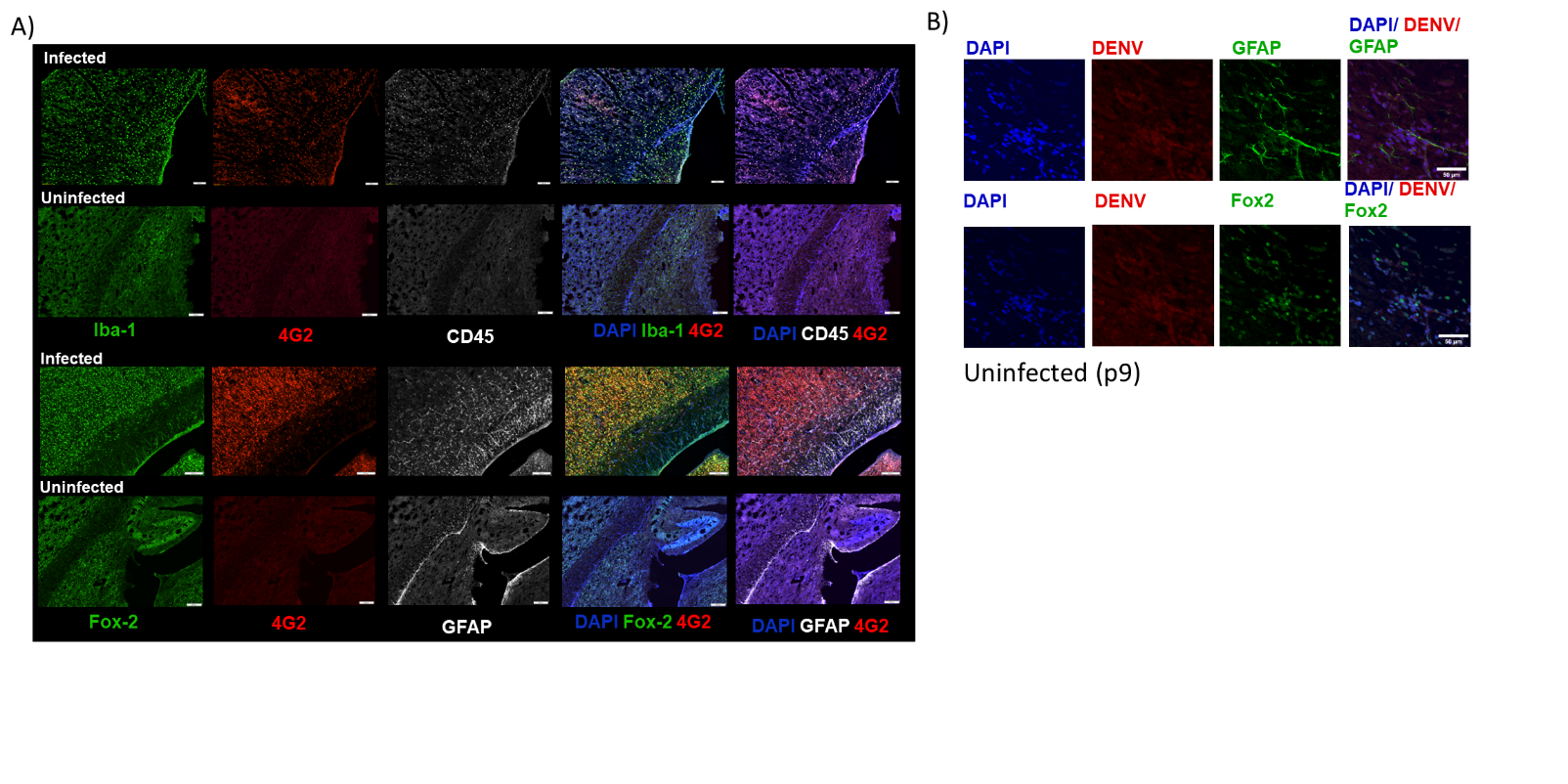
**

**Supplementary Figure 2**

**
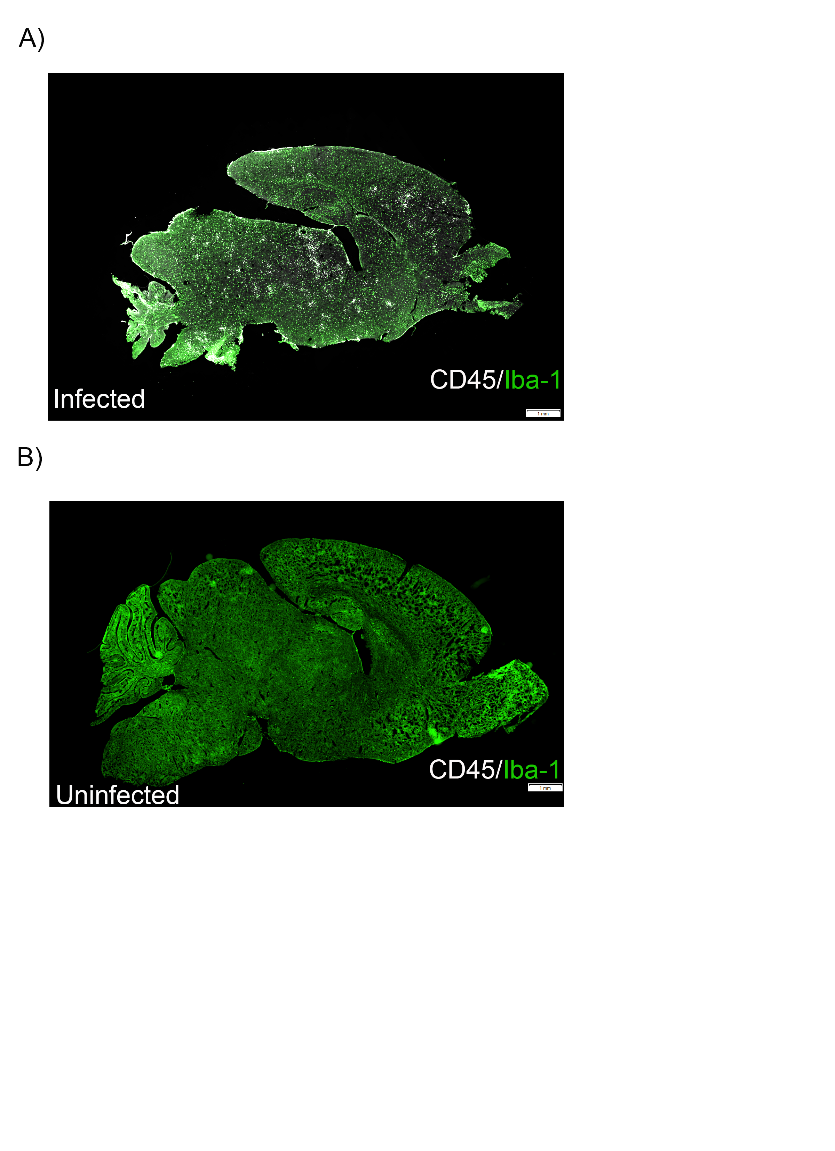
**

**Supplementary Figure 3**

**
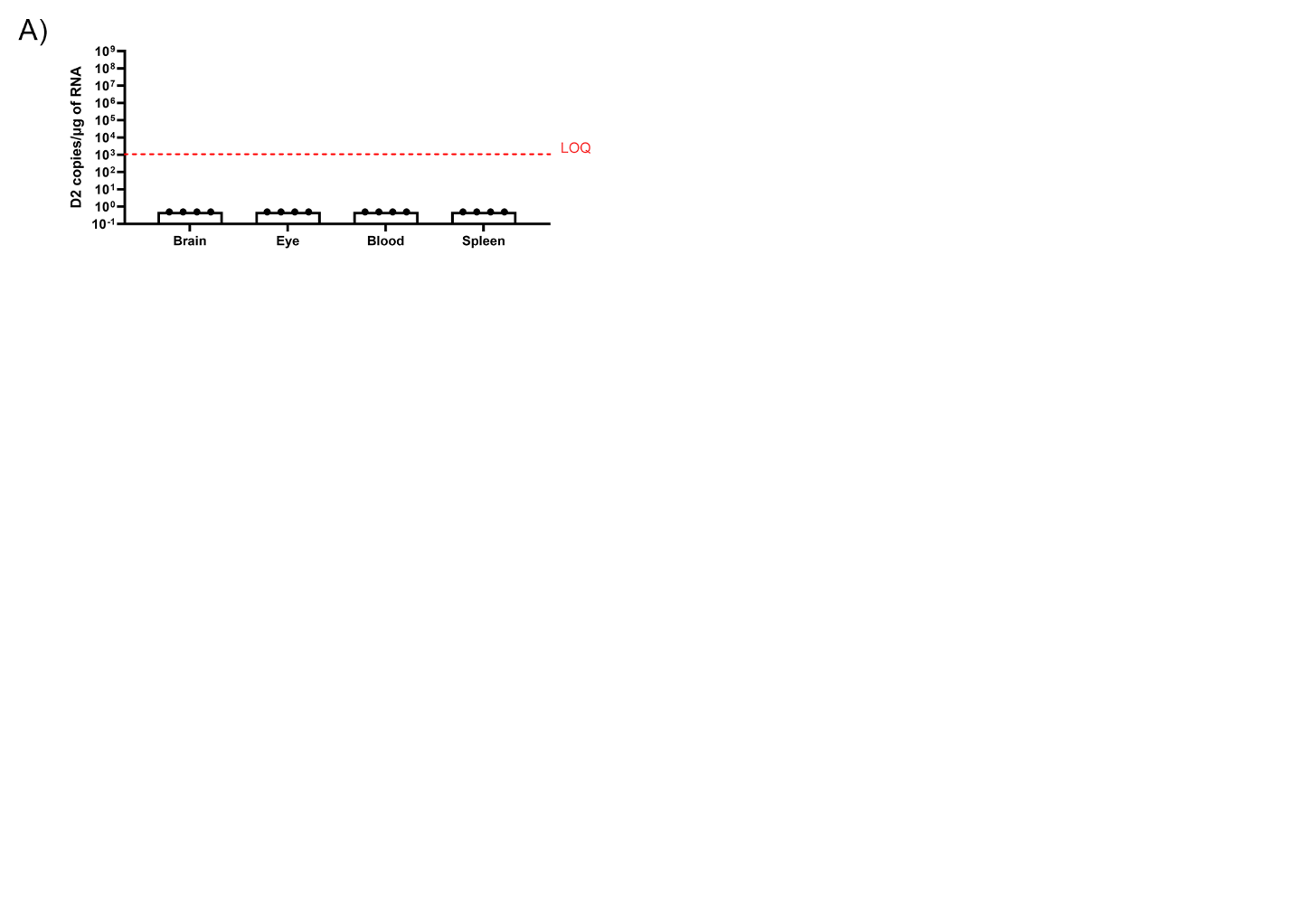
**

**Supplementary Figure 4**

Supplement: Supplementary Figures.docx [file TEMI_A_2477668_SM8146.docx]
